# Gate-tunable negative differential resistance in multifunctional van der Waals heterostructure

**DOI:** 10.1038/s41598-026-68365-1

**Published:** 2026-08-24

**Authors:** Richa Mitra, Konstantina Iordanidou, Naveen Shetty, Md Anamul Hoque, Anushree Datta, Alexei Kalaboukhov, Julia Wiktor, Sergey Kubatkin, Saroj Prasad Dash, Samuel Lara-Avila

**Affiliations:** 1 Department of Microtechnology and Nanoscience, Chalmers University of Technology, 412 96 Gothenburg, Sweden; 2 Department of Physics, Chalmers University of Technology, 412 96 Gothenburg, Sweden; 3 Materials Physics-Oslo, SINTEF Industry, 0314 Oslo, Norway; 4 Laboratoire Matériaux et Phénomenes Quantiques, Université Paris-Cité, CNRS, 75013 Paris, France; 5 Laboratoire de Physique des Solides, Université Paris-Saclay, CNRS, 91405 Orsay, France; 6 National Physical Laboratory, Hampton Road, Teddington, TW11 0LW UK; 7 Present Address: VTT Technical Research Centre of Finland Ltd, Espoo, Finland

**Keywords:** Engineering, Materials science, Nanoscience and technology, Physics

## Abstract

Two-dimensional (2D) semiconductors have emerged as exciting candidates for the development of low-power and multifunctional computing applications, thanks to their qualities such as layer-dependent band gap tunability, high carrier mobility, and excellent electrostatic control. Here, we explore a pair of 2D semiconductors with nearly broken-gap (Type-III-like) band alignment and demonstrate a highly gate-tunable p-MoTe_2_/n-SnS_2_ heterojunction with multifunctional behavior. Employing a dual-gated asymmetric device geometry, we unveil its functionality as both a forward and backward rectifying device. Moreover, we observe a highly gate-tunable negative differential resistance (NDR), with a gate-coupling efficiency of *η* ≃ 0.5 and a peak-to-valley ratio of ∼ 3 down to 150 K. By employing density functional theory, we determine that the observed NDR is dominated by valence band-to-valence band tunneling, while additional interband tunneling contributions arise at higher bias. The combination of tunneling driven transport and gate controllability of NDR opens the pathway for realizing gate-tunable 2D material-based neuromorphic and energy-efficient electronics.

**Supplementary Information:**

The online version contains supplementary material available at https://doi.org/10.1038/s41598-026-68365-1.

## Introduction

The quest for enhanced computational power, energy efficiency, and data processing capabilities continues to fuel the demand for novel materials and computational paradigms^1–4^. Tunnel field effect transistors (TFETs) emerge as exciting candidates to realize the long-standing promise of energy-efficient computation, using band-to-band tunneling (BTBT)^5–10^ as the primary carrier injection mechanism, as opposed to conventional thermal injection of field-effect transistors (FETs). This would allow to circumvent inherent fundamental constraints of FETs, because BTBT allows for sharper turn ON/OFF of the transistors and reduce sub-threshold swing (SS = $\:\frac{\partial\:{V}_{G}}{\partial\:{I}_{SD}}$) below the thermionic limit of 60 mVdec^− 1^ at room temperature^4–10^(with I_SD_ the source-drain current, and V_G_ the gate voltage). Importantly, TFETs can exhibit negative differential resistance (NDR) under specific conditions^11–26^, which opens avenues for the implementation of multi-valued logic in emerging applications such as high frequency oscillators^27^, artificial intelligence^28^ and neuromorphic computing^29^.

In recent years, two-dimensional (2D) materials^1–4^ are being explored to boost the performance of TFETs and to bypass challenges often encountered when implementing TFETs with bulk semiconductors: the absence of dangling bonds on 2D crystals ensures a pristine, bond-free surface, and allows for the creation of atomically clean and sharp interfaces in 2D van der Waals (vdW) heterostructures. Moreover, 2D material TFETs provide an edge over their three-dimensional counterparts, as reduced screening in two dimensions facilitates better electrostatic control of devices. With the plethora of 2D materials available at our disposal, it is crucial to carefully select combinations of materials that act as source and the channel of the TFET. A common design rule is to choose a narrow bandgap p-type source and a wider bandgap n-type channel. This enhances the density of tunneling carriers, enabling efficient carrier injections from the source to the channel. The larger bandgap of the channel material suppresses off-state leakage, thereby improving the device’s ON/OFF current ratio. Several heterojunctions implemented following these prescriptions have been demonstrated^6,11–22^. Recent theoretical works have sparked interest into unconventional pairs of 2D semiconductors^29–31^ having broken (Type-III alignment) or nearly broken-gap band alignment, suitable for TFET operation, as it facilitates efficient band-to-band-tunneling, that would result in high on-state current, low subthreshold swing, reduced power consumption and faster logic operations.

Here we explore one such unconventional pair of 2D semiconductors in the light of tunable nearly broken-gap (Type-III-like) band alignment by combining a few layers of p-MoTe_2_ and n-SnS_2_, which are predicted to form a naturally nearly broken-gap band alignment^32–34^. We demonstrate multifunctional behavior of the junction, showing tunable forward to reverse rectifying behavior with gate voltage and observe gate-tunable NDR^12,17,19,20,26–28,35–36^ at temperatures down to 150 K. Our first-principles calculations based on density functional theory (DFT) shed light on our experimental observations and provide insight in device optimization pathways.

## Results: theoretical prediction

**Fig. 1:**
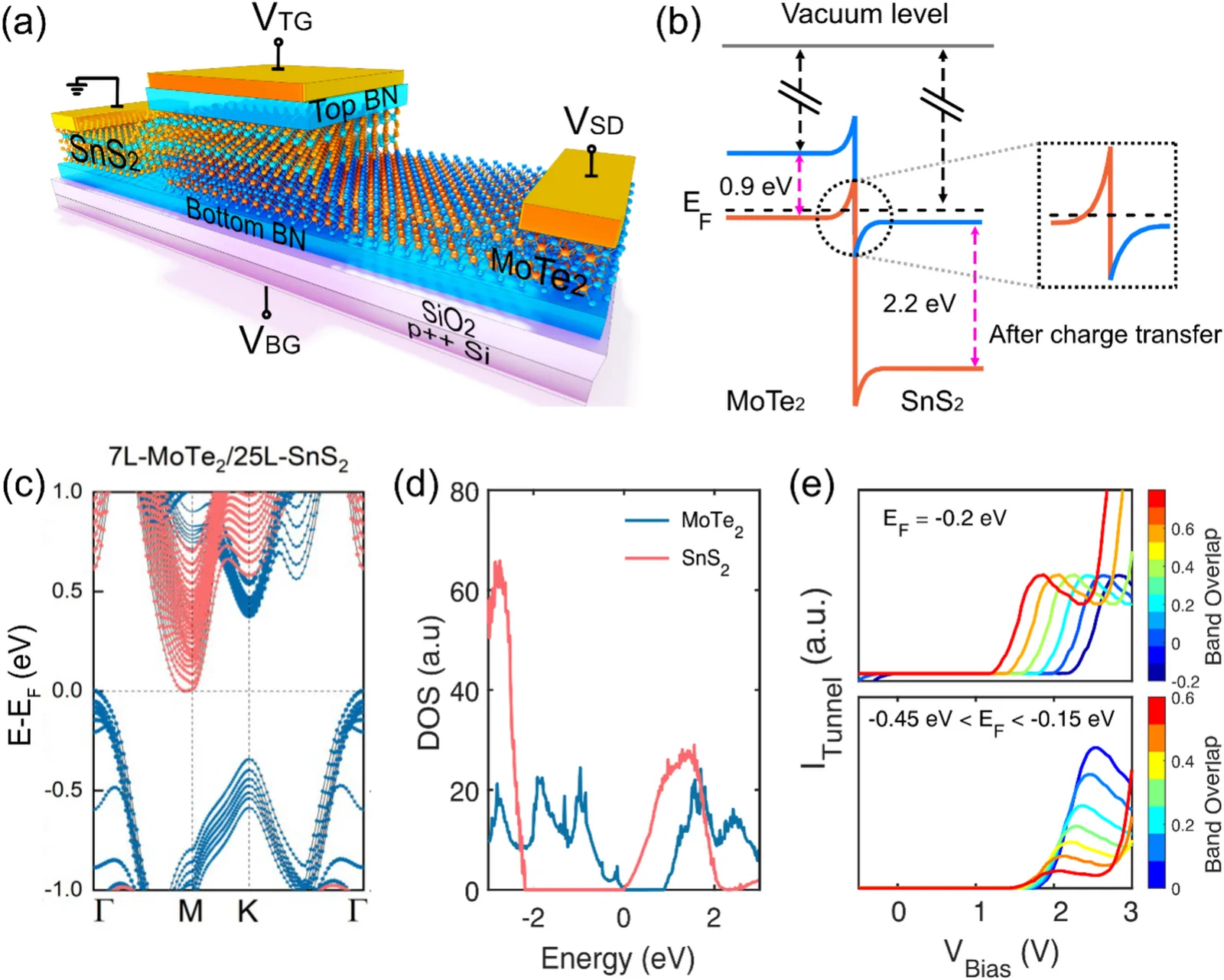
Type-III heterojunction with MoTe_2_/SnS_2_: (**a**) Device schematic of a dual gated vertical heterojunction of MoTe_2_ and SnS_2_ on a Si/SiO_2_ substrate. Backgate V_BG_ and topgate voltages V_TG_ are applied across the SiO_2_ substrate and top hBN respectively to change the carrier density. Biasing configuration is also shown, where V_SD_ is applied to MoTe_2_, SnS_2_ is grounded. (**b**) Nearly broken-gap (type -III-like) band alignment of MoTe_2_ and SnS_2_ after forming the junction. Band bending appears as a result of charge transfer from MoTe_2_ to SnS_2_ (KPFM results in Supplementary Info section S4). The enlarged view at the interface illustrates the nearly broken-gap alignment with a small overlap between the MoTe₂ valence band and SnS₂ conduction band. (**c**) Band structure from DFT calculations for 7-layer MoTe_2_/25-layer SnS_2_ heterojunction showing MoTe_2_ VB maxima aligning with SnS_2_ CB minima. (**d**) Partial density of states of MoTe_2_ and SnS_2_ from DFT. (**e**) Tunnel current (I_Tunnel_) calculated from Eq. (1) using DFT DOS, plotted w.r.t theoretically applied bias voltage (V_Bias_). I_Tunnel_ are estimated using DFT DOS for different band alignment configurations: (Upper panel) The extent of band overlaps i.e., the broken-gap alignment is varied (as indicated by the color bar, in units of eV), while the Fermi energies are held constant across the junction (E_Fm_ = E_Fs_ = −2 eV). In this configuration, I_Tunnel_ remains unchanged while V_peak_ shifts systematically. (Bottom panel) Simultaneously varying the Fermi energies (e.g. −0.45 eV *<* E_F_ *<* −0.15 eV) and the band overlap results in systematic changes in both the magnitude of I_Tunnel_ and the position of V_peak_ (SI section S2).

A schematic of our proposed device, a MoTe_2_/SnS_2_ heterojunction, is shown in Fig. 1a. In this device architecture, MoTe_2_ has bandgap around ≈ 0.9 eV and acts like a source material, whereas SnS_2_ having a larger bandgap (≈ 2.2 eV) and higher electron affinity serves as a channel material. Figure 1b illustrates the schematic of a nearly broken-gap band alignment in vacuum. A qualitative schematic illustrating charge-transfer-induced band bending at the interface has been shown in the Supplementary Information (Fig. S4). To support the band alignment, we performed first-principles DFT calculations with van der Waals corrections, which reveal a nearly broken-gap configuration at the MoTe₂/SnS₂ interface. Figure 1c shows the corresponding DFT-computed band structure, highlighting the overlap between the MoTe₂ valence band (VB) and the SnS₂ conduction band (CB) density of states. While Fig. 1c presents the results for a specific layer thickness combination, our calculations^31,32,34^ show that the nearly broken-gap alignment is a robust feature across various MoTe₂/SnS₂ heterostructure thicknesses (see Supplementary info SI Section S1). The first-principles calculations also allow us to anticipate the device performance of the MoTe₂/SnS₂ heterojunction. Specifically, we use the partial density of states (DOS) of MoTe₂ and SnS₂ to compute the tunneling current using the following expression^11^:

1 $$\:{I}_{Tunnel}=\:{\int\:}_{{E}_{Fm}}^{{E}_{Fs}}{DOS}_{{MoTe}_{2}}\left(E\right)\times\:{DOS}_{{SnS}_{2}}(E-e{V}_{Bias})\times\:\left[{f}_{{MoTe}_{2}}\right(E)-{f}_{{SnS}_{2}}(E-e{V}_{Bias}\left)\right]dE$$

Here $\:{DOS}_{{MoTe}_{2}}$ ($\:{DOS}_{{SnS}_{2}}$) and $\:{f}_{{MoTe}_{2}}$ ($\:{f}_{{SnS}_{2}}$) represent the DOS and the Fermi distribution function of MoTe_2_ (SnS_2_) respectively. In Fig. 1d we plot the partial DOS of the two materials with zero ‘band overlap’ which indicates that the VB maxima of MoTe_2_ aligns with CB minima of SnS_2_ at Energy = 0. We define negative (positive) ‘band overlap’ to the scenario when SnS_2_ CB minima lie lower (higher) in energy than VB maxima of MoTe_2_. We also retain the usual convention of biasing where the positive (negative) $\:{V}_{Bias}$ shifts the SnS_2_ bands towards higher (lower) energy with respect to the MoTe_2_ bands (detail calculation shown in SI Section S2).

Our calculations of the theoretical tunneling current ($\:{I}_{Tunnel}$) through the MoTe_2_/SnS_2_ heterojunction as a function of the bias voltage ($\:{V}_{Bias}$) indicate that such device will display tunable NDR features. Figure. 1(e) shows $\:{I}_{Tunnel}$ for two different band alignment configurations. The upper panel refers to a scenario where the Fermi levels remain constant across the heterojunction while the extent of the band overlap is varied, transitioning from a partially staggered (Type-II) to a nearly broken-gap configuration (Type-III-like). In this case, the voltage corresponding to the NDR peak (V_peak_) shifts to lower bias values as the band overlap increases. In contrast, under the second configuration where both the Fermi levels and band overlap vary systematically across the junction, both the peak current magnitude and the peak voltage increase. These results indicate that the NDR response in MoTe₂/SnS₂ heterojunctions is robust and can be modulated through electrostatic gating. While the nearly broken-gap alignment permits interband VB-CB tunneling in principle, our calculations with overlapping DOS under applied bias indicate that the dominant contribution to the observed NDR originates from tunneling between valence band states of MoTe₂ and SnS₂, as discussed further in Supplementary Section S2. We note that the calculations shown in Fig. 1c were performed for a specific combination of layer thicknesses. Although thickness dependent variations in the band gap, screening, and work function can quantitatively modify the band overlap, the observed gate-tunable NDR in our MoTe₂/SnS₂ devices is consistent with a nearly broken-gap/type-III-like alignment. Direct thickness-resolved measurements of the band offsets would require additional techniques such as KPFM or optical spectroscopy and are beyond the scope of the present work.

## Results: device fabrication

**Fig. 2:**
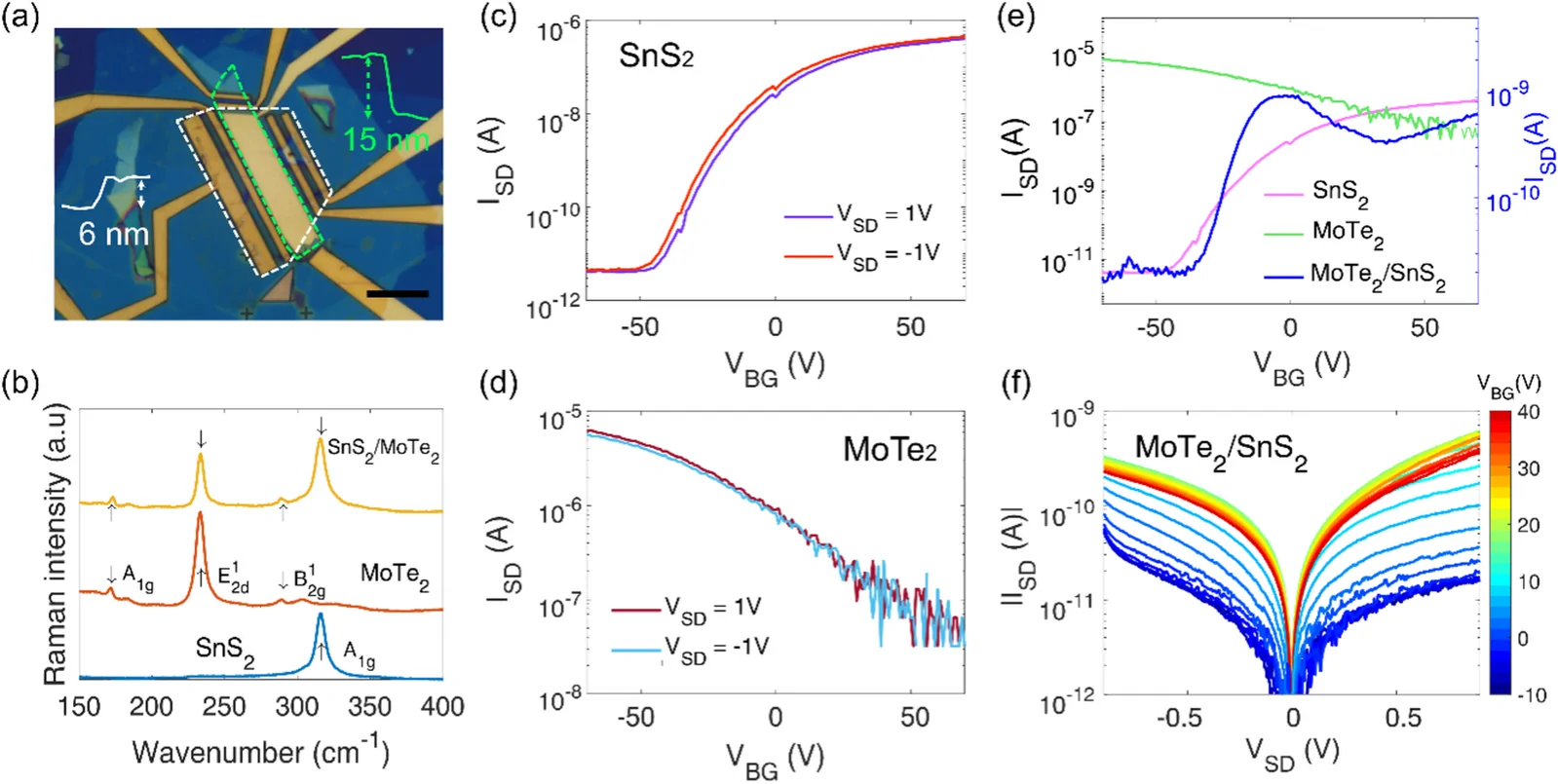
Device characterisation from backgated device D1: (**a**) Optical micrograph of the fabricated dual-gated MoTe₂/SnS₂ heterojunction device. The scale bar represents 10 μm. The inset shows the AFM height profiles of the MoTe₂ and SnS₂ flakes. (**b**) Material characterization of the individual layers using Raman spectroscopy. We used a 532 nm laser source for acquiring the Raman signal of the individual flakes as well as the junction of the device. The Raman spectra show the characteristic A₁_g_ phonon mode of SnS₂^37^ at 315 cm^−1^. For MoTe₂^38^, Raman peaks are observed at wave numbers 233 cm^−1^, 173 cm^−1^, and 289 cm^−1^, which correspond to the in-plane E^1^_2g_, out-of-plane A_1g_, and B^1^_2g_ modes, respectively. The MoTe_2_/SnS_2_ junction carries Raman signals from both materials (indicated by arrows), as shown in the yellow curves. Transfer characteristic curves of SnS_2_ (**c**) and MoTe_2_ (**d**) measured at room temperature for V_SD_ = ± 1 V and plotted in logarithmic scale for backgate voltages from − 70 V to 70 V. (**e**) Transfer curves of the MoTe_2_/SnS_2_ junction (blue, right axis) plotted together with MoTe_2_ (green, left axis), SnS_2_ (pink, left axis) for V_SD_ = 1 V. (**f**) Output characteristic i.e. I_SD_ vs. V_SD_ curves for the MoTe_2_/SnS_2_ junction for a range of backgate voltages (shown in colorbar) showing modulation of both forward and reverse bias current with V_BG_.

To fabricate devices, we stack 2D material flakes into heterostructures using a polymer-based hot pickup technique. After exfoliating the flakes onto Si/SiO_2_ and identifying suitable ones, the polymer-based pick-up at elevated temperatures ensures residue-free interfaces, which is critical for device fabrication. To enhance the interface quality, we perform vacuum annealing followed by an atomic force microscopy (AFM) scan to ensure surface cleanliness. We use Ti (5 nm)/Au (70 nm) and Pd (15 nm)/Au (60 nm) for making ohmic contacts to SnS_2_ and MoTe_2,_ respectively. This contact engineering approach results in high p-type doping in few-layer MoTe_2_ and n-type doping in SnS_2_. For the dual-gated geometry, hBN/MoTe_2_/SnS_2_/hBN stacks are fabricated by adding a hBN layer (≈ 20 nm) over the junction. We have fabricated 5 devices according to the above-mentioned process (all fabrication and characterization details in SI section S3, S4). However, we present extensive data for one backgated device (D1) and two dual-gated devices (D2, D3) (shown in Fig. 2a). During device fabrication and before electrical measurements we characterize the thickness/morphology and chemical composition of individual flakes using atomic force microscopy (AFM) and Raman spectroscopy, respectively. The AFM topography reveals that MoTe_2_ and SnS_2_ flakes are multilayer (in the range of 4–9 layers for the former one and 12–25 layers for the later, respectively as shown in the inset of Fig. 2a). Raman analysis in Fig. 2b shows typical modes for each layer present in the junction, with pronounced modes at 233 cm^− 1^ for MoTe_2_ and at 315 cm^− 1^ for SnS_2_.

## Backgated device

To understand the performance of the MoTe_2_/SnS_2_ junction, we first present the response of individual flakes for a backgated device D1 at room temperature. Figure 2c,d show the transfer characteristics (source-drain current I_SD_ vs. backgate voltage V_BG_) of SnS_2_ and MoTe_2_ flakes respectively at a fixed source-drain voltage, V_SD_ = ±1 V.

SnS_2_ alone exhibits n-type FET behavior with ∼ 5 orders magnitude increase in current from OFF to ON state. In comparison, MoTe_2_ exhibits p-type conduction with high current density; its lower ON/OFF ratio can be attributed to its higher intrinsic doping and to its small band gap, which allows simultaneous transport of both holes and electrons. The nearly identical responses for both bias polarities indicate ohmic contact formation, as further confirmed by the linear I–V characteristics (see SI Section S5A). We further estimate the effective Schottky barrier at the Pd/Au-MoTe_2_ and Ti/Au-SnS_2_ contacts from the temperature dependent transport measurements of a representative backgated device to examine the carrier injection at the metal-semiconductor interfaces (see SI Section S5B and Fig. S5B). The extracted barriers provide insight into the contact properties of the individual flakes. Next, Fig. 2e shows the transfer characteristics of the individual flakes SnS_2_ and MoTe_2_ plotted on the left axis, along with the corresponding response of the MoTe_2_/SnS_2_ junction (plotted on the right axis). We find that the current across the junction shows a non-monotonic behavior with respect to the applied backgate voltage, which we attribute to the creation of a p/n density profile along the junction as a result of screening of the applied electric fields. Figure 2f completes the device characterization, depicting the I-V characteristic curves of the MoTe_2_/SnS_2_ junction in logarithmic scale for a range of backgate voltages. We observe that the source-drain current systematically increases with the applied backgate voltage, indicating a reduction in junction resistance. This increase is evident for both forward and reverse bias, showing no significant rectification behavior. Additionally, the absence of NDR suggests that the junction remains in a regime of limited band overlap and strong electrostatic screening, preventing the formation of a broken-gap alignment necessary for NDR onset^12,17,20,35,39^. This highlights the importance of employing dual-gated geometries to enable more effective control over band alignment and carrier distribution across the junction.

## Dual-gated device

**Fig. 3:**
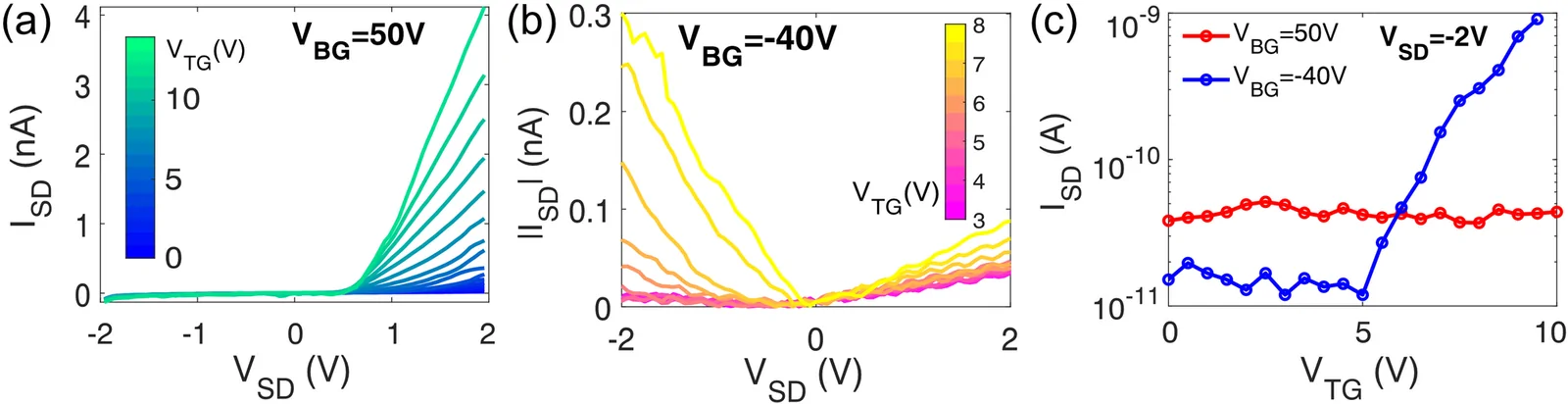
Gate-dependent current rectification: Output curves for opposite polarities of the backgate voltages of (**a**) 50 V and (**b**) − 40 V showing rectification at forward and reverse bias respectively. (**c**) Topgate response of the junction current at reverse bias (V_SD_ = − 2 V) comparing rectification at − 40 V to 50 V backgate voltage (Data for V_SD_ = 2 V is shown in SI section S6).

To enhance the junction tunability and explore its performance as TFET device, we implement a topgate electrode using hBN as the dielectric in addition to the global bottom gate. With the topgate primarily modulating the carrier concentration of the SnS_2_ flake, our measurements reveal multifunctional transport behavior of the MoTe_2_/SnS_2_ heterojunction under opposite backgate polarities^35,39^. Figure 3a,b demonstrate that, at certain combinations of bottom and topgate voltages (V_TG_) our device acts as a rectifier in both forward bias (FB) and reverse bias (RB) conditions. In Fig. 3a,b, the I–V curves are plotted on a linear scale for a range of V_TG_, as indicated in the colorbars. Figure 3a shows at positive backgate voltage (50 V) the FB drain current increases significantly with rising V_TG_, while RB current remains low and unaffected, because increasing the topgate voltage raises the SnS_2_ Fermi energy creating more charge carriers for conduction in FB, while the RB current is limited by interband tunneling, which remains suppressed due to insufficient overlap between the DOS. In contrast, Fig. 3b shows that for a negative polarity of backgate voltage (− 40 V), pronounced current rectification is observed for negative source drain bias, arising from charge carriers tunneling from the filled VB of MoTe_2_ to empty CB band of SnS_2_^35,36^. We note that the rectification mechanism described in this section and the NDR discussed later corresponds to different backgate regimes and should not be attributed to the same tunneling process. At V_BG_ = − 40 V, the strong p-doping of MoTe_2_ favors VB-CB interband tunneling responsible for the enhanced reverse-bias current. In contrast, the NDR observed at V_BG_ = − 10 V occurs under a different electrostatic band alignment and is predominantly governed by VB-VB tunneling, as supported by our DFT calculations and DOS-overlap analysis, with an additional VB-CB contribution possible at higher bias. Figure 3c summarizes the rectification behavior by showing the change of source-drain current with topgate voltage, at different polarities of the backgate voltage for a fixed negative bias (− 2 V). The observed rectification can be qualitatively understood by examining the transport characteristics of the individual constituent flakes (Fig. 2c,d)^5,6,26^. The gate-dependent rectification behavior reflects changes in the relative carrier availability in MoTe_2_ and SnS_2_ under different backgate polarities, resulting in suppressed reverse-bias current when MoTe_2_ is depleted and enhanced tunneling when it is p-doped. A qualitative band-alignment schematic, provided in Supplementary Section S6 (Fig. S6), is included only as a guiding illustration.

## Negative differential resistance

Having described the gate-tunable rectification in MoTe_2_/SnS_2_, we now turn to the observation of NDR in dual-gated devices. Our theoretical model indicates that NDR in the MoTe_2_/SnS_2_ heterostructure emerges when the band alignment approaches a nearly broken-gap configuration, where enhanced band overlap leads to efficient valence band-to-valence band tunneling, while additional interband tunneling contributions arise at higher bias. Such a regime is realized in our heterostructure for specific combinations of top- and backgate voltages.

**Fig. 4:**
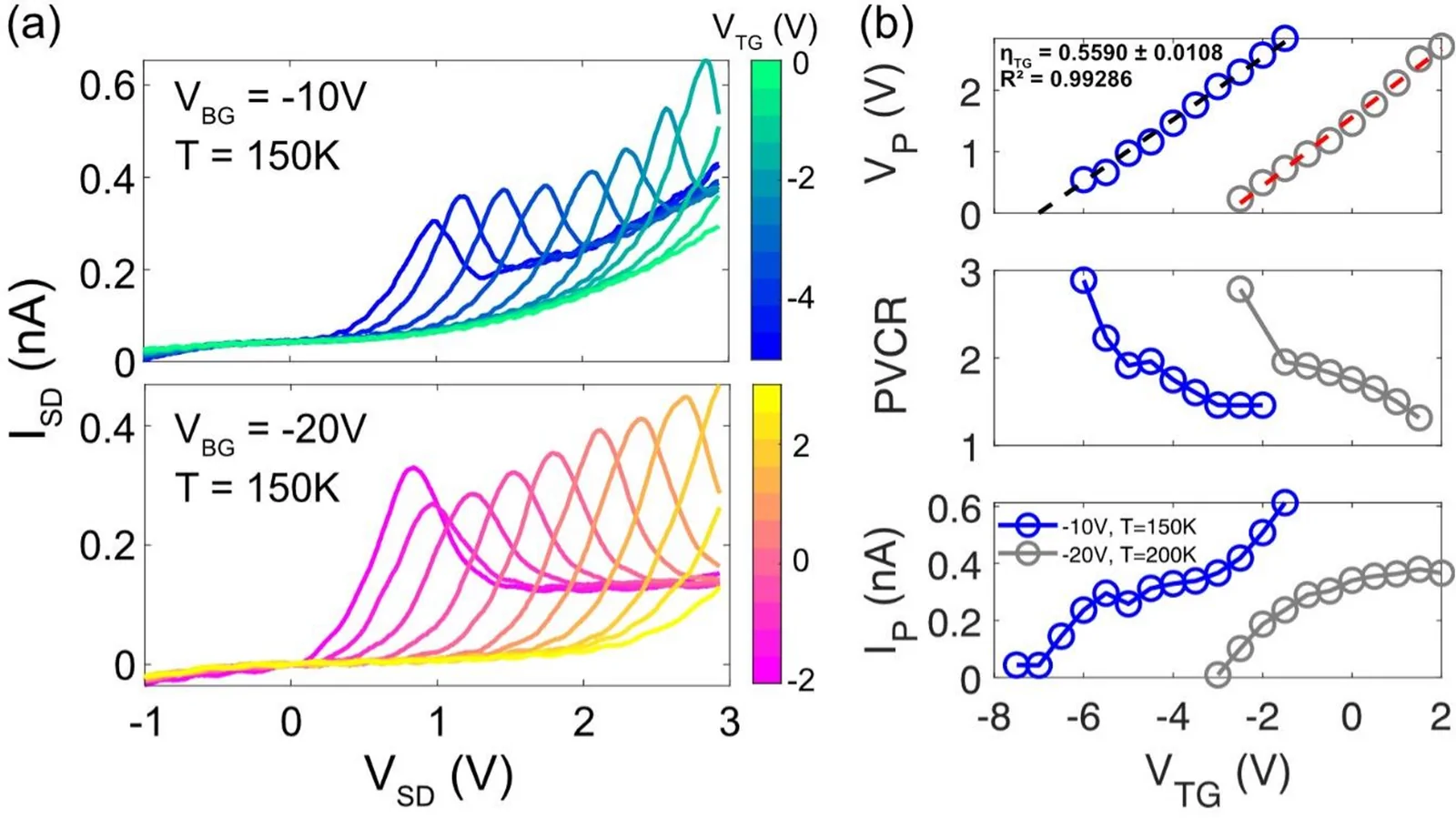
Characterization of gate-tunable negative differential resistance in dual gated devices: (**a**) Output curves of the dual-gated device D2 showing NDR response at V_BG_ = − 10 V (top panel), at V_BG_ = − 20 V (bottom panel) measured at T=150 K. Colorbars represent topgate voltages. (**b**) (top panel) V_P_ (bias voltage at which the NDR peak appears) vs. V_TG_ plot. Linear fits yield top-gate coupling efficiencies of η_TG_ = 0.5291 ± 0.0114 (R^2^ = 0.99633) over V_TG_ = − 6 to − 1.5 V (black dashed line) and η_TG_ = 0.5590 ± 0.0108 (R^2^ = 0.99286) over V_TG_ = − 2.5 to 2 V (red dashed line) for the blue and gray datasets, respectively. (Middle panel) Peak-to-valley current ratio (PVCR = $\:\frac{{I}_{Peak}}{{I}_{Valley}}$) vs. V_TG_ plot. (Bottom panel) Peak current I_P_ vs. V_TG_. The blue and the gray circles (curves) represent data from device D2 measured at V_BG_ = − 10 V at 150 K and V_BG_ = − 20 V at 200 K, respectively.

In Fig. 4a, upper panel, we present I-V curves at a fixed backgate voltage V_BG_ = − 10 V, using the topgate voltage V_TG_ as parameter (indicated in the colorbar). We observe that, for each I–V curve the current changes non-linearly with the bias voltage, increasing from negative voltages until it reaches a local maximum at certain bias (peak voltage, V_P_). The effect of the topgate is twofold. First, the topgate allows to tune the position of the NDR peak voltage V_P_: as V_TG_ reduces from 0 V, V_P_ systematically shifts to a lower bias voltage. Second, we also observe orderly change in the peak current (= I_P_) with the application of topgate voltage. For comparison, the bottom panel of Fig. 4a shows similar gate-tunable NDR features are observed for a different backgate voltage, V_BG_ = − 20 V (see SI section S7 for NDR in other experimental conditions)^12,17,19,20,35–37^.

To gain more insight into the observed NDR feature, we study the topgate dependence of the transport characteristics of our dual-gated devices. The top panel of Fig. 4b shows the NDR peak voltage (V_P_) as a function of V_TG_ for two representative datasets. In both cases, V_P_ varies linearly with V_TG_, demonstrating strong top-gate control of the NDR peak position. We define the top-gate coupling efficiency as $\:{{\upeta\:}}_{TG}=\left|\frac{\varDelta\:{V}_{P}}{{\varDelta\:V}_{TG}}\right|.\:$ For V_BG_ = − 10 V at T = 150 K, a linear fit over V_TG_ = − 6 to − 1.5 V yields $\:{{\upeta\:}}_{TG}$ = 0.5291 ± 0.0114 with R^2^ = 0.99633. For V_BG_ = − 20 V at T = 200 K, fitting over V_TG_ = − 2.5 to 2 V gives $\:{{\upeta\:}}_{TG}$ = 0.5590 ± 0.0108 with R^2^ = 0.99286. Thus, both datasets consistently yield $\:{{\upeta\:}}_{TG}$ ≈ 0.5. This corresponds to an approximately 0.5 V shift in the NDR peak for every 1 V change in topgate voltage. The high $\:{{\upeta\:}}_{TG}$ indicates strong capacitive coupling and efficient top-gate control of the nearly broken-gap band alignment^35,36^. The corresponding back-gate coupling efficiency, η_BG_, extracted for device D3, is discussed separately in Section S8 of the Supplementary Information. The considerably lower η_BG_ ≈ 0.1 compared with η_TG_ ≈ 0.55 indicates that the top gate provides substantially stronger electrostatic control over the NDR peak position and the corresponding band alignment. Similar gate-induced modulation of the tunneling barrier has been reported in other 2D vdW devices, highlighting the importance of electrostatic control in determining charge injection and transport across such junctions^40^.

Figure 4(b) (middle panel) shows the V_TG_ dependence of the Peak-to-Valley Current Ratio, PVCR =$\:\frac{{I}_{Peak}}{{I}_{Valley}}$, a key performance indicator of NDR devices which are comparable to or better than previously reported values in other 2D heterostructures^12,17,20,25^. In our device, we find the maximum PVCR to be ∼ 3–4. Figure 4b (bottom panel) shows the peak current as a function of the topgate voltage plotted for the similar V_BG_ and temperature combinations. The observed increase in peak current with topgate voltage indicates enhanced tunneling probability, which likely arises from reduced band misalignment and more efficient carrier injection across the junction.

**Fig. 5:**
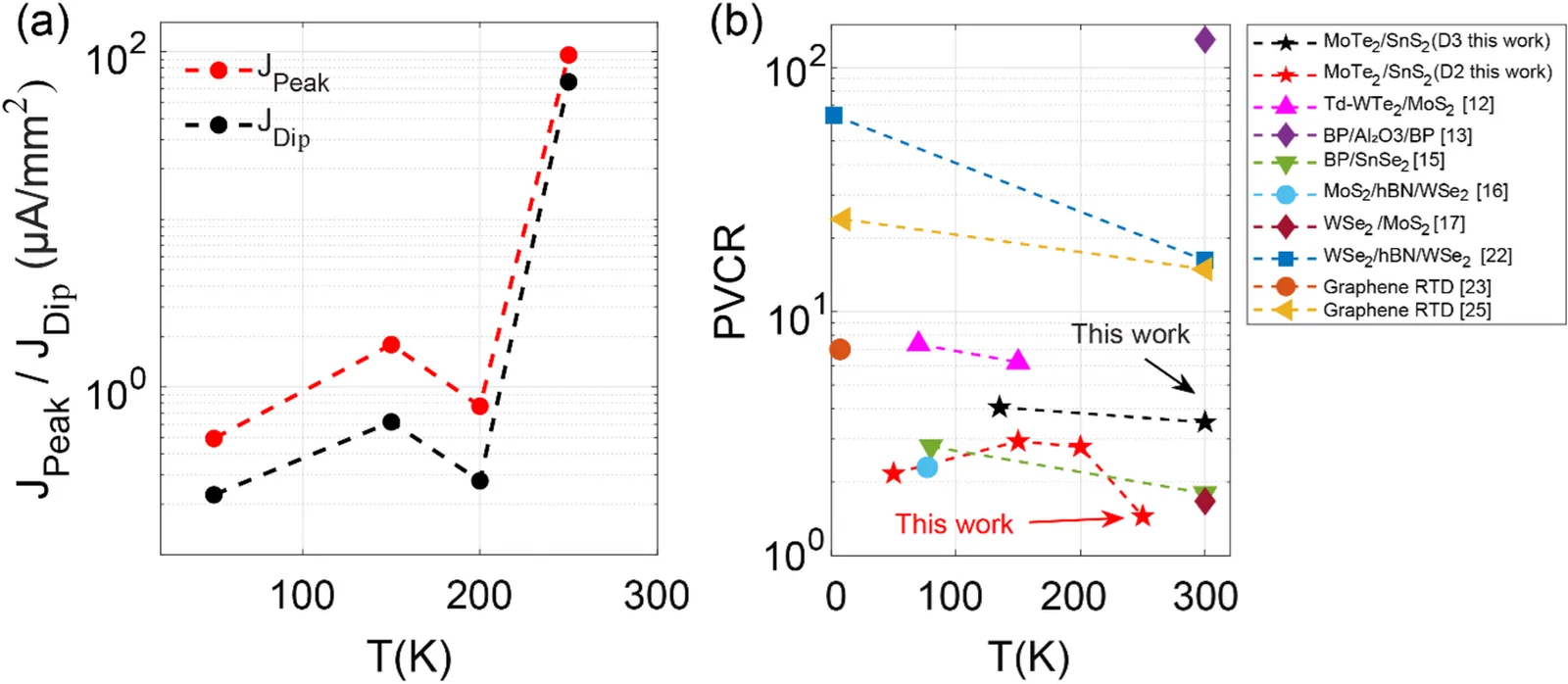
Temperature dependence: (**a**) Temperature dependence of the peak and dip current densities in our MoTe_2_/SnS_2_ devices (normalized by device area 132 μm^2^ and plotted in units of µA/mm^2^ for device D2). (**b**) Plot of peak-to-valley-ratio (PVCR) vs. temperature for various van der Waals heterostructure NDR devices including this work (red and black markers). This plot highlights the temperature sensitivity and performance trends across different 2D material systems.

## Discussion

We now discuss the performance limits of our TFET devices by examining the temperature dependence of the observed NDR in our MoTe_2_/SnS_2_ heterojunctions and how it impacts the PVCR values. Figure 5a shows the temperature dependence for both the peak ($\:{J}_{Peak}$) and dip( $\:{J}_{Dip}$) current densities for Device D2. Correspondingly, the variation of $\:{J}_{Peak}$ and $\:{J}_{Dip}$ leads to a certain temperature dependence in PVCR, shown in Fig. 5b which is compared to other contemporary van der Waals NDR devices. The maximum experimentally observed PVCR of ≈ 4 at T = 150 K in our MoTe_2_/SnS_2_ heterostructure is already comparable to other reported van der Waals systems. However, these high values do not persist at either low or high temperatures.

A possible scenario for the low-temperature regime (T < 150 K) is that the reduced phonon population limits phonon-assisted tunneling arising from the momentum mismatch between the valence band maximum of MoTe_2_ and the conduction band minimum of SnS_2_^37,38^. The resulting reduction in inelastic tunneling events can suppress carrier transport across the junction. Recent studies on layered TaIrTe_4_^41^ have demonstrated substantial electron-phonon coupling, highlighting the relevance of electron-phonon interactions to the temperature-dependent electronic properties of vdW materials. At lower temperatures, reduced thermal broadening is also expected to decrease the valley current, which can enhance the PVCR. However, the simultaneous reduction in phonon-assisted tunneling and effective DOS overlap may suppress the overall tunneling current, contributing to the observed non-monotonic temperature dependence.

While reduced phonon-assisted processes may limit NDR at lower temperatures, different transport mechanisms become increasingly important as the temperature is raised. In the higher temperature limit (T > 200 K), we observe that both $\:{J}_{Peak}$ and $\:{J}_{Dip}$ increase; however, the PVCR reduces due to larger increase in the $\:{J}_{Dip}$ values. This behavior suggests the emergence of additional leakage mechanisms such as thermionic emission or trap-assisted tunneling becoming more prominent at higher temperatures and conceal the NDR signature by increasing the valley current. Moreover, effects like thermal broadening of energy distribution also play significant role in reducing the effective band-to-band-tunneling, thereby further degrading the PVCR. It is in the intermediate temperature regime, T ~ 150 K, the thermally activated carrier injection is optimized while leakage through thermionic and trap-assisted channels remains suppressed, giving rise to a maximum PVCR^12,22^.

Compared to other vdW heterostructures, BP-based and graphene-based systems exhibit the highest PVCR values. In BP-based devices, this is largely due to suitability of band alignment engineering, strong anisotropic carrier transport, and high-quality interfaces achieved in homojunctions that minimize leakage and non-resonant tunneling^9,11,13–15,26^. In contrast, graphene-based resonant tunneling diodes show high PVCR due to momentum-conserving tunneling across atomically flat barriers like hBN, enabling sharp energy filtering and resonant transmission^23–25,34–39^. Following such examples, the PVCR in MoTe_2_/SnS_2_ heterojunctions could be further enhanced by optimizing band overlap and minimizing parasitic leakage. This could be achieved by for instance, inserting a thin dielectric spacer (e.g., hBN) between semiconducting layers^11,17,21,22^ to reduced interface disorder and suppress defect-assisted tunneling, which together sharpen the resonance condition and reduce valley current.

## Conclusion

In summary, we demonstrated a multifunctional dual-gated vdW heterojunction by combining a few layers of MoTe_2_ and SnS_2_. The junction exhibits strong gate tunability, transitioning from forward to reverse rectifying behavior at opposite backgate voltages, with a rectification ratio exceeding two orders of magnitude. At intermediate gating conditions, the device exhibits Esaki diode behavior, showing robust negative differential resistance up to temperatures of 200 K. The NDR features are highly tunable via the topgate voltage, with the peak voltage exhibiting a linear dependence on topgate bias (topgate-coupling efficiency ~ 0.5), and a PVCR approaching ∼4 at 150 K. Using DFT calculations, we find that transport is dominated by valence band-to-valence band tunneling, with an additional valence band-to-conduction band tunneling contribution at higher bias, while gate tunability originates from changes in band overlap and Fermi-level alignment. The gate-tunable features of the NDR curves are attributed to a combined effect of band overlap modulation and Fermi level tuning induced by the topgate. The strong gate coupling enables effective manipulation of broken-gap alignment in the heterostructure. These results establish MoTe_2_/SnS_2_ as a versatile platform for exploring tunable tunneling transport and demonstrate how NDR can serve as a sensitive probe of band alignment in 2D heterostructures. Our findings underscore the importance of material choice, band structure engineering, and dual-gating schemes for advancing energy-efficient logic and multifunctional nanoelectronic devices.

## Methods

We fabricate the heterojunctions using a standard exfoliation and 2D material stacking process. MoTe_2_, SnS_2_, and hBN crystals are exfoliated using blue Nitto tape or Scotch tape over small pieces of PDMS sheets. Suitable flakes of the materials are identified and selected under an optical microscope. Using a micro-manipulator equipped with x, y, and z precision stages, as well as rotation and heater configurations, we transfer hBN, MoTe_2_, and SnS_2_ onto Si/SiO_2_ substrates in that order. After the stacking process, the devices are vacuum annealed at 120 °C for 2 h at a pressure of approximately 2 × 10^− 6^ mbar. This step helps achieve a residue-free interface.

For contact fabrication, the samples are spin-coated with a bilayer resist followed by e-beam lithography. The source and drain contacts are fabricated in a two-step process, where we pattern the contacts on MoTe_2_ and SnS_2_ flakes separately using e-beam lithography, followed by e-gun evaporation of metal and liftoff in hot acetone. We use Pd/Au (15 nm/60 nm) and Ti/Au (5 nm/70 nm) as the metal combinations for MoTe_2_ and SnS_2_, respectively. For dual-gated devices, we transfer another hBN layer on top of the junction and define the topgate using a similar patterning process.

All samples are wire-bonded and loaded into a 4 K wet cryostat for characterization and measurement. We use a variable gain current amplifier, FEMTO DHPCA-100, for applying biasing voltage and current measurement, and a Keithley 2400 for backgating and topgating purposes.

## Supplementary Information

Below is the link to the electronic supplementary material.

Supplementary Material 1

## Data Availability

The datasets generated and analyzed in this study are available from the corresponding authors upon reasonable request. Details of the theoretical calculations are provided in the Supplementary Information.
