# Correction: Gate-tunable negative differential resistance in multifunctional van der Waals heterostructure

**DOI:** 10.1038/s41598-026-70585-4

**Published:** 2026-09-09

**Authors:** Richa Mitra, Konstantina Iordanidou, Naveen Shetty, Md Anamul Hoque, Anushree Datta, Alexei Kalaboukhov, Julia Wiktor, Sergey Kubatkin, Saroj Prasad Dash, Samuel Lara-Avila

**Affiliations:** 1 Department of Microtechnology and Nanoscience, Chalmers University of Technology, 412 96 Gothenburg, Sweden; 2 Department of Physics, Chalmers University of Technology, 412 96 Gothenburg, Sweden; 3 Materials Physics-Oslo, SINTEF Industry, 0314 Oslo, Norway; 4 Laboratoire Matériaux et Phénomenes Quantiques, Université Paris-Cité, CNRS, 75013 Paris, France; 5 Laboratoire de Physique des Solides, Université Paris-Saclay, CNRS, 91405 Orsay, France; 6 National Physical Laboratory, Hampton Road, Teddington, TW11 0LW UK; 7 Present Address: VTT Technical Research Centre of Finland Ltd, Espoo, Finland

Correction to: *Scientific Reports* https://doi.org/10.1038/s41598-026-68365-1, published online 24 August 2026

In the original version of this Article, Saroj Prasad Dash and Samuel Lara-Avila were omitted as corresponding authors. Correspondence and requests for materials should also be addressed to saroj.dash@chalmers.se and samuel.lara@chalmers.se

Furthermore, an affiliation was omitted for Richa Mitra. Their correct affiliations are:

‘Department of Microtechnology and Nanoscience, Chalmers University of Technology, 412 96 Gothenburg, Sweden.’ and present address: ‘VTT Technical Research Centre of Finland Ltd, Espoo, Finland.’

The original Article has been corrected.

