# Supplementary material for: Gate-tunable negative differential resistance in multifunctional van der Waals heterostructure
Source: Sci Rep. 2026 Aug 24;16:26526. doi: 10.1038/s41598-026-68365-1 (PMC13503671; doi:10.1038/s41598-026-68365-1)
Supplement: Supplementary file 1 [file 41598_2026_68365_MOESM1_ESM.docx]

**Supplementary Information: Gate-tunable negative differential resistance in multifunctional van der Waals heterostructure**

Richa Mitra^1,∗,§^ Konstantina Iordanidou^2^*^,^*^3^, Naveen Shetty^1^, Md Anamul Hoque^1^, Anushree Datta^4^*^,^*^5^, Alexei Kalaboukhov^1^, Julia Wiktor^2^, Sergey Kubatkin^1^, Saroj Prasad Dash^1,†^ Samuel Lara-Avila^1^*^,^*^6‡^

^1^Department of Microtechnology and Nanoscience, Chalmers University of Technology, SE-412 96, Gothenburg, Sweden

^2^Department of Physics, Chalmers University of Technology, SE-412 96, Gothenburg, Sweden

^3^Materials Physics-Oslo, SINTEF Industry, NO-0314, Oslo, Norway

^4^Laboratoire Mat´eriaux et Ph´enomenes Quantiques, Universit´e Paris-Cit´e, CNRS, 75013, Paris, France

^5^Laboratoire de Physique des Solides, Universit´e Paris-Saclay, CNRS, 91405, Orsay, France

^6^National Physical Laboratory, Hampton Road, Teddington, TW11 0LW, United Kingdom

∗[richa@chalmers.se](mailto:richa@chalmers.se)

^§^ Present address: VTT Technical Research Centre of Finland Ltd, Espoo, Finland

^†^[saroj.dash@chalmers.se](mailto:saroj.dash@chalmers.se)

^‡^[samuel.lara@chalmers.se](mailto:samuel.lara@chalmers.se)

# S1. Band structure calculation with different layer thickness

To investigate the thickness dependent electronic structure and band alignment of MoTe₂/SnS₂ heterostructures, density functional theory (DFT) calculations were performed using the Vienna ab initio simulation package (VASP). The electron-ion interaction was described by PAW potentials with valence electron configurations of 4d^10^5s^2^5p^2^ for Sn, 3s^2^3p^4^ for S, 4s^2^4p^6^4d^5^5s^1^ for Mo, and 5s^2^5p^4^ for Te. Periodic boundary conditions were applied and a vacuum space thicker than 15 ˚A was included in the vertical direction to remove fictitious interactions between adjacent slabs. For both optimizations and electronic structure calculations van der Waals corrections were considered through the optB86b-vdW functional, and for all calculations the kinetic energy cutoff was set to 500 eV. Owing to the relatively low lattice mismatch between MoTe_2_ and SnS_2_ layers, heterostructures are built by simply stacking the corresponding unit cells. For our models, the strain is evenly shared within MoTe_2_ and SnS_2_, and upon relaxations, both the atomic positions and the lattice constants are optimized. As a first step, we build heterostructures consisting of single-layer MoTe_2_ and SnS_2_, with six high-symmetry stacking patterns. We find that the heterostructure for which top-layer S atoms are above Te atoms and bottom-layer S atoms are above Mo atoms, is the energetically favorable one. This stacking pattern is adopted for building thicker models, namely 4L-MoTe_2_/12L-SnS_2_, 6L-MoTe_2_/29L-SnS_2_, 7L-MoTe_2_/29L-SnS_2_, 14L- MoTe_2_/12L-SnS_2_, 7L-MoTe_2_/25L-SnS_2_, and 36L-MoTe_2_/25L-SnS_2_, which correspond to the experimentally observed thicknesses. Concerning the electronic properties, using standard DFT, we find that single-layer MoTe_2_ is a semiconductor with a direct band gap of 1.14 eV, and by increasing the number of layers the band gap decreases. On the other hand, single-layer SnS_2_ is an indirect gap semiconductor of 1.51 eV and smaller gaps are also observed for thicker SnS_2_ layers. As shown in Fig. S1 all studied heterostructures exhibit the nearly broken-gap band alignment which is promising for realizing tunneling transistors. Here we note that, for all studied heterostructures there is a small overlap between the high-lying valence states of MoTe_2_ and the low-lying conduction states of SnS_2_ apart from 36L-MoTe_2_/25L-SnS_2_ heterostructure where a small effective gap is observed.

**Figure S1:** Band alignment of MoTe_2_/SnS_2_ heterostructure for different layer thicknesses. All the plots indicate nearly broken-gap band alignment.

# S2. Calculation of Tunnel current using DFT DOS

In this section we demonstrate the details of tunnel current (I_Tunnel_) calculations from the density of states (DOS) extracted from the density functional theory (DFT). We base our modeling on the partial DOS of MoTe_2_ and SnS_2_ computed within DFT. Since DFT yields very good predictions of the shape of the DOS but often fails to reproduce the experimental band gaps of materials, we also apply a scissor shift to the calculated valence and conduction band to match experimental band gaps of 0.9 eV for bulk MoTe_2_ [1,2] and 2.2 eV for bulk SnS_2_ [3] respectively. To study the effect of the size of the broken band gap, we also shift the DOS of the two materials in respect to each other and place the Fermi levels to match experimental observations of NDR in the present study.

Fig. S2(a) and (b) show the equilibrium band alignment for two representative cases: (a) $E_{F}^{m}=E_{F}^{s}=-0.45$eV, corresponding to zero band overlap, and (b) $E_{F}^{m}=E_{F}^{s}=-0.15$eV, corresponding to a band overlap of 0.6 eV. In all panels, the density of states (DOS) of MoTe₂ (blue curve) and SnS₂ (orange curve) is plotted as a function of energy $E$ under equilibrium conditions for different band alignment scenarios. For all calculations, we use the DFT-calculated partial density of states (DOS) of MoTe₂ and SnS₂ after formation of the heterojunction. In all equilibrium plots, positive (negative) energy corresponds to conduction band (valence band) states. The zero overlap band alignment is defined by alignment of the SnS₂ conduction band minimum (CBM) with the MoTe₂ valence band maximum (VBM). A positive (negative) band overlap corresponds to the SnS₂ CBM lying higher (lower) in energy relative to the MoTe₂ VBM. The tunneling current $I_{\mathrm{Tunnel}}$ is calculated using the following expression:

$I_{Tunnel}= \int_{E_{Fm}}^{E_{Fs}} {DOS}_{{MoTe}_{2}}(E)\times{DOS}_{{SnS}_{2}}(E-eV_{Bias})\times[f_{{MoTe}_{2}}(E)-f_{{SnS}_{2}}(E-eV_{Bias})]dE$ (1)

Here ${DOS}_{{MoTe}_{2}}$ (${DOS}_{{SnS}_{2}}$) and $f_{{MoTe}_{2}}$ ($f_{{SnS}_{2}}$) represent the DOS and the Fermi distribution function of MoTe_2_ (SnS_2_) respectively. Fig. S2(a) and (c) show the band alignment for scenario (1) with $E_{F}^{m}=E_{F}^{s}=-0.45$eV, corresponding to zero band overlap (or an effective gap of 0 eV), under biasing conditions of $V_{\mathrm{Bias}}=0$V and 2.2 V, respectively. From these plots, we observe that the integrand in Eq. (1), namely the DOS *product* (green curves), becomes non-zero only when there is a finite overlap between the relevant bands and depends on the relative positions of the Fermi energies of the two materials. The corresponding integrated $I$–$V$ characteristics are shown in Fig. S2(f), where the colored arrows indicate the applied bias voltages, mentioned in the figure caption.

Fig. S2(c) and (d) show that VB–VB tunneling contributes dominantly to the total current, while at higher bias voltages an additional contribution arises from overlap between the SnS₂ valence band and the MoTe₂ conduction band, as shown in Fig. S2(e). These calculations indicate that the dip in current appears when the SnS₂ valence band maximum aligns with the MoTe₂ bandgap. As Fermi energies evolve with increasing band overlaps, the NDR peak current is correspondingly modulated, accompanied by a systematic shift of the peak position toward different bias voltages.

**Figure S2:** **Calculation of I_Tunnel_:** Plots of partial DOS of MoTe_2_ (blue), SnS_2_ (orange), E_Fm_ (yellow), E_Fs_ (purple) and the integrand of the equation 1 (green) vs. energy for two equilibrium conditions (V_Bias_ = 0V) at **(a)** E_Fm_ = E_Fs_ = −0.45 eV with band overlap of 0 eV and **(b)** E_Fm_ = EFs = −0.15 eV with band overlap of of 0.6 eV. Similar plots showing broken band alignment when bias voltage is applied, i.e. at **(c)** E_Fm_ = E_Fs_ = −0.45 eV with band overlap of 0 eV at 2.2V, and for E_Fm_ = E_Fs_ = −0.15 eV with band overlap of 0.6 eV at **(d)** 2V and **(e)** 3V. **(f)** I_Tunnel_ vs V_Bias_ plot for the mentioned scenarios. The colored arrows correspond to different bias voltages as shown in plot (c)-(e): pink→(c), cyan→(d) and green→(e)

# S3. Device fabrication

In this section, we describe the steps of device fabrication which involves exfoliation and stacking of hexagonal boron nitride (hBN), MoTe_2_ and SnS_2_, followed by fabrication of electrical contacts and topgate probes. We start by exfoliating thin flakes of MoTe_2_, SnS_2_ and hBN from their respective bulk crystals using blue Nitto tape or scotch tape. The exfoliated flakes are transferred from the tapes to separate PDMS sheets (we cut 4mmx 4mm pieces of PDMS and stick them on transparent glass slides). Flakes with suitable shapes and thicknesses of different material are identified under the optical microscope carefully. After identifying suitable hBN, MoTe_2_ and SnS_2_ flakes under optical microscope, they are sequentially transferred onto a separate Si/SiO_2_ substrate having predefined alignment markers. The substrates are always cleaned with hot acetone followed by IPA and baked at 120°C for 15 minutes to remove organic contaminants and the adsorbed water. We use a transfer setup equipped with x, y and z precision alignment and heating stage for assembling the stacks at ambient condition. The step-by-step procedure of exfoliation and dry transfer technique has been shown schematically in Fig. S3 (a). After stacking hBN/MoTe_2_/SnS_2_, we anneal the final stack inside a vacuum anneal chamber at 120°C for 2 hours at a pressure of *~* 2 *×* 10*^−^*^6^ mbar. Annealing aids removing any organic residues trapped inside the van der Waals layer which helps improve the interface quality [4]. After that, we spin coat the substrate with bilayer resist (Copolymer MMA/ARP) followed by baking the resist over hotplate at 150°C for 5 minutes. We pattern the source and drain contacts on MoTe_2_ and SnS_2_ flakes separately using e-beam lithography followed by e-gun evaporation of metal and liftoff. We use the metal combinations of Pd/Au (15nm/60nm) and Ti/Au (5nm/70nm) for MoTe_2_ and SnS_2_, respectively. We choose high work function Pd (Φ = 5*.*6eV) [5, 6] which forms ohmic contact with MoTe_2_ and helps efficient injection of p-type carriers into the channel. We find Titanium (Φ = 4*.*33eV) [7] to be suitable for injecting electrons into SnS_2_. Finally, liftoff is performed in the hot acetone to remove the excess metal. For the dual-gated devices, we further stack additional hBN to cover the junction area following the aforementioned dry transfer method. After that, a topgate is defined over the overlap junction of MoTe_2_/SnS_2_ using e-beam lithography followed by Ti/Au (15nm/80nm) deposition. For the topgate, we choose the hBN thickness to be *~* 15 *−* 20 nm (AFM height measurement shown in Fig. S3b and c) .

**Figure S3:** **Device fabrication: (a)** Step by step process for 2D material exfoliation and dry transfer method. AFM height measurement of top hBN used as the topgate dielectric in devices **(b)** D2 and **(c)** D3.

# S4. Charge transfer and work function calculation using Kelvin Probe Force Microscopy

**Figure S4: KPFM measurement of the MoTe₂/SnS₂ heterojunction.** **(a)** Schematic band alignment of the MoTe₂/SnS₂ heterojunction before and after junction formation, illustrating band bending due to charge transfer from MoTe₂ to SnS₂. **(b)** Two-dimensional KPFM map of the MoTe₂/SnS₂ device. The scale bar corresponds to 4 µm. Blue and green dashed boundaries indicate MoTe₂ and SnS₂ regions, respectively, while the solid lines mark the line scans used for work function extraction. **(c)** Work function ($\Phi$) profiles extracted along the line scans shown in (b). SnS₂ on MoTe₂ exhibits a reduced work function compared to SnS₂ on SiO₂, indicating electron transfer from MoTe₂ to SnS₂.

Due to the difference in Fermi energies between SnS₂ and MoTe₂, charge transfer is expected to occur when the two flakes come into contact in the heterostructure, leading to Fermi level equilibration and interfacial band bending, as schematically illustrated in Fig. S4(a) (before and after charge transfer). We used Kelvin probe force microscopy (KPFM) to experimentally probe this charge redistribution and to quantify the work functions of SnS₂ and MoTe₂. In KPFM, the contact potential difference ($V_{CPD}$) is measured between the work function of the tip ($\Phi_{tip}$) and that of the sample ($\Phi_{sample}$), following:

$V_{CPD}= \frac{(\Phi_{tip}-\Phi_{Sample})}{e}$

(2)

where, *e* is the elementary charge. To determine the absolute work function of the sample, we first calibrated the tip by measuring $V_{CPD}$ on highly oriented pyrolytic graphite (HOPG), which has a known work function of $\Phi_{HOPG}$ = 4.48 eV. The tip work function is then given by:

$\Phi_{tip}=eV_{CPD}^{HOPG}+\Phi_{HOPG}$	(3)

In our calibration, $V_{CPD}$ = 1.0 V for the Pt–Ir coated tip on HOPG, yielding $\Phi_{tip}$ ≈ 5.48 eV. Fig. S4(b) shows a line profile of the CPD signal measured across the MoTe₂/SnS₂ heterojunction. A clear contrast is observed between SnS₂ on MoTe₂ and SnS₂ on the SiO₂ substrate, indicating a spatial variation in surface potential. The corresponding work function ($\Phi$) profiles extracted along the two-line scans marked in Fig. S4(b) are shown in Fig. S4(c). We find the work function of MoTe₂ on SiO₂ to be approximately 4.2 eV, consistent with reported literature values. Notably, SnS₂ on MoTe₂ exhibits a lower work function of approximately 3.6 eV, indicating electron transfer from MoTe₂ to SnS₂, consistent with charge redistribution across the heterojunction interface.

# S5A. Linear I-V curves

# Figure S5A: Ohmic nature of the contacts: (a) Output characteristics of SnS₂ and (b) MoTe₂ showing source-drain current ($I_{\mathrm{SD}}$) as a function of source-drain voltage ($V_{\mathrm{SD}}$) measured from −2 V to +2 V for a range of backgate voltages. The linear $I_{\mathrm{SD}}\text{-}V_{\mathrm{SD}}$behavior confirms the ohmic nature of the contacts in both materials.

Figure S5 (a) and (b) show the source-drain current (I_SD_) vs. bias voltage (V_SD_) applied across the individual flakes. The arrows show series of curves for a set of backgate voltages from -70V to 70V in the step of 10V, measured at 300K. For SnS_2_, the I_SD_-V_SD_ curves are linear within ±2V. However, for MoTe_2_, the current tends to behave semi-linearly at higher bias voltages.

# S5B. Effective Schottky Barrier Calculations for MoTe_2_/Pd and SnS_2_/Ti Contacts

# Figure S5B: Effective Schottky barrier extraction for MoTe_2_/Pd and SnS_2_/Ti contacts using temperature dependent transport measurements. (a) Transfer characteristics ($I_{SD}$–$V_{BG}$) of the SnS_2_ measured at $V_{SD}=1$V over the temperature range of 70–280 K. (b) Transfer characteristics of the MoTe_2_ measured at $V_{SD}=2$V over 70–300 K temperature range. The MoTe_2_ and SnS_2_ flakes belong to a backgated device similar to device D1 used in the main text. (c,d) Richardson plots of $\ln\left( \frac{I_{SD}}{T^{2}} \right)$as a function of $1000/T$ for different gate voltages extracted from the temperature dependent measurements of SnS_2_ and MoTe_2_, respectively. The dashed lines represent linear fits performed according to the Richardson equation: $\boldsymbol{I}_{\boldsymbol{SD}}\boldsymbol{=}AA^{*}T^{2}\exp(-\frac{\phi_{B}}{k_{B}T})$ where $A^{*}$ is the Richardson constant, $T$is the temperature, $k_{B}$ is the Boltzmann constant, and $\phi_{B}$ is the effective Schottky barrier height. The extracted values correspond to effective activation barriers obtained from thermionic transport analysis. (e) Gate dependent effective Schottky barrier heights extracted from the linear fits in panels (c) and (d). The purple diamonds represent the extracted barrier height of SnS_2_ ($\phi_{\mathrm{Sn}S_{2}}$, left y-axis), while the green hexagons represent the extracted barrier height of MoTe_2_ ($\phi_{\mathrm{MoT}e_{2}}$, right y-axis). The bottom and top x-axes correspond to the gate voltage ranges used for SnS_2_ and MoTe_2_ respectively.

# To estimate the carrier injection barriers at the contacts, temperature dependent transport measurements were performed on backgated MoTe_2_ and SnS_2_ devices similar to device D1. The effective Schottky barrier height ($\phi_{B}$) was extracted using Richardson analysis by fitting the temperature dependence of the current according to $\boldsymbol{I}_{\boldsymbol{SD}}\boldsymbol{=}AA^{*}T^{2}\exp(-\phi_{B}/k_{B}T)$ as shown in Fig. S5B (c) and (d). The extracted values shown in Fig S5B (e) represent effective activation barriers associated with carrier injection and do not correspond to direct TLM contact resistance measurements.

# S6. Forward and reverse rectifying characteristics

**Figure S6:** **Forward and reverse rectifying characteristics of the MoTe_2_/SnS_2_ heterojunction: (a,b)** Source-drain current ($I_{SD}$) as a function of topgate voltage ($V_{TG}$) measured at backgate voltages $V_{BG}=50$V (red) and $-40$V (blue), for (a) $V_{SD}=-2$V and (b) $V_{SD}=+2$V, demonstrating reverse- and forward-rectifying behavior, respectively. **(c,d)** Schematic band diagrams illustrating the transport mechanisms under reverse bias (RB) and forward bias (FB) conditions for (c) $V_{BG}=50$V (red dashed boxes) and (d) $V_{BG}=-40$V (blue dashed boxes). The orange and blue shaded regions represent the occupied density of states in MoTe₂ and SnS₂, respectively, while the unshaded regions represent states. The arrows indicate the dominant electron transport pathways, and the crossed arrows indicate energetically unfavorable or suppressed tunneling. At $V_{BG}=50$V, the MoTe₂ channel is highly depleted, resulting in a negligible change in the reverse bias current despite modulation of the SnS₂ electron density by the topgate. In contrast, at $V_{BG}=-40$V, p-doping of MoTe₂ enhances the availability of occupied valence band states, promoting interband tunneling under reverse bias and increased carrier transport under forward bias.

In this section, we describe the rectification behavior of the MoTe₂/SnS₂ heterostructure using a tentative band alignment picture. Figs. S6(a) and (b) show the I_SD_ vs V_TG_ plots for reverse bias (-2V) and forward bias (2V) respectively. The red and blue traces correspond to rectification at 50V and -40V backgate voltages. The rectification process can be understood by considering the responses of individual flakes (Fig. 2c, d of main manuscript together with the proposed band alignment schematics shown in Figs. S6(c) and (d). At positive backgate voltage (V_BG_ = 50V), MoTe_2_ is depleted of p-type carriers resulting in reduced band bending at the interface. Consequently, the charge carrier tunneling from the MoTe₂ valence band to the SnS₂ conduction band is strongly suppressed, leading to negligible reverse bias current. Although applying a positive topgate voltage increases the carrier density in SnS₂ and shifts E_Fs_ into the conduction band, the lack of carriers in MoTe₂ limits the junction current, rendering it largely insensitive to topgate modulation (as shown by the red curve in Fig. S6 (a)).

In contrast, at negative backgate voltage (V_BG_ = −40V), MoTe_2_ becomes strongly p-doped which leads to: (a) E_Fm_ shifting deeper into the valence band, (b) E_Fs_ approaching the conduction band edge due to enhanced charge transfer and increased band bending. This band alignment facilitates a finite RB tunneling current from the MoTe₂ valence band to the SnS₂ conduction band. Furthermore, increasing the topgate voltage shifts E_Fs_ further into the conduction band, enhancing the available density of states and resulting in a pronounced increase in reverse bias current, as indicated by the blue trace in Fig. S6(a). Under forward bias conditions, the reduced band bending favors carrier transport dominated primarily by thermionic emission over the junction barrier. This process depends strongly on the SnS₂ DOS and initial band alignment, making it more sensitive to gate modulation when MoTe₂ is depleted.

**S7. Gate-tunable NDR at** V_BG_ = *−*50**V, 50K**

**Figure S7:** **Gate-tunable NDR:** Topgate tunability of NDR signal observed in device D2 at *V*_BG_ = *−*50V at 50K. This plot shows the NDR peak shifting towards higher bias at higher *V*_TG_ with negligible modulation in peak current.

**Figure S8:** **Backgate tunability of NDR of device D3: (a-c)** Source–drain current ($I_{\mathrm{SD}}$) as a function of bias voltage ($V_{\mathrm{SD}}$) measured at different backgate voltages ($V_{\mathrm{BG}}$), showing the evolution of the NDR feature at (a) 300 K, $V_{\mathrm{TG}}=0$V, (b) 135 K, $V_{\mathrm{TG}}=0$V, and (c) 135 K, $V_{\mathrm{TG}}=2$V. **(d)** Backgate-coupling efficiency ($\eta_{BG}$) extracted from linear fits to the NDR peak voltage ($V_{P}$) as a function of $V_{\mathrm{BG}}$ at 300 K and 135 K. **(e)** NDR peak current ($I_{P}$) and **(f)** peak-to-valley current ratio (PVCR) plotted as a function of $V_{\mathrm{BG}}$ at 300 K and 135 K. The pink and black markers correspond to data at 300K and 135K respectively.

# S8. Backgate tunability of NDR of device D3

We present the backgate tunability of NDR measured in the dual-gated device D3 at 300K and at 135K. Figures S8 (a-c) show I_SD_ - V_SD_ characteristics measured at 300K and 135K for V_TG_ =0V, and at 135K for V_TG_ =2V. A clear evolution of the NDR feature with backgate voltage ($V_{\mathrm{BG}}$) is observed under all measurement conditions. The backgate coupling efficiency $\eta_{BG}= |\frac{\Delta V_{P}}{{\Delta V}_{BG}}|$ is extracted from the linear dependence of the NDR peak voltage (V_P_) on $V_{\mathrm{BG}}$ as shown in Fig. S8 (d). At 135 K, a linear fit over the range V_BG_ = 20–40 V yields $\eta_{BG}$ = 0.0830 ± 0.0086 with R² = 0.96847, while at 300 K, fitting over V_BG_ = 46–50 V gives $\eta_{BG}$ = 0.1271 ± 0.0080 with R² = 0.99592. These values are considerably smaller than the top-gate coupling efficiency, $\eta_{TG}$≈ 0.55, obtained for device D2, indicating weaker electrostatic control of the NDR peak position by the back gate. This difference is consistent with the weaker capacitive coupling through the relatively thicker backgate dielectric. Figure S8(e) shows a systematic variation of the peak current, I_P_, with V_BG_, while the corresponding peak-to-valley current ratio (PVCR) reaches values of up to ~4, as shown in Fig. S8(f).

# References

# [1] Ruppert, C., Aslan, B. & Heinz, T. F. Optical properties and band gap of single- and few-layer MoTe₂ crystals. Nano Lett. 14, 6231–6236 (2014).

# [2] Koo, B. et al. Vertical-tunneling field-effect transistor based on MoTe₂/MoS₂ 2D–2D heterojunction. J. Phys. D: Appl. Phys. 51, 475101 (2018).

# [3] Schlaf, R., Lang, O., Pettenkofer, C. & Jaegermann, W. Band lineup of layered semiconductor heterointerfaces prepared by van der Waals epitaxy: Charge transfer correction term for the electron affinity rule. J. Appl. Phys. 85, 2732–2753 (1999).

# [4] Jain, A. et al. Minimizing residues and strain in 2D materials transferred from PDMS. Nanotechnology 29, 265203 (2018).

# [5] Kim, C. et al. Fermi level pinning at electrical metal contacts of monolayer molybdenum dichalcogenides. ACS Nano 11, 1588–1596 (2017).

# [6] Aftab, S. et al. Formation of an MoTe₂-based Schottky junction employing ultra-low and high-resistive metal contacts. RSC Adv. 9, 10017–10023 (2019).

# [7] Lv, C. et al. Electrical contact barriers between a three-dimensional metal and layered SnS₂. ACS Appl. Mater. Interfaces 12, 15830–15836 (2020).
